# Political Attitude and Fertility: Is There a Selection for the Political Extreme?

**DOI:** 10.3389/fpsyg.2018.02343

**Published:** 2018-11-27

**Authors:** Martin Fieder, Susanne Huber

**Affiliations:** Department of Evolutionary Anthropology, Faculty of Life Sciences, University of Vienna, Vienna, Austria

**Keywords:** evolution, number of children, political attitude, behavior genetics, liberal–conservative

## Abstract

There is growing evidence that human ideology as well as social and political attitudes also have a genetic basis. In case of some genetic predisposition of political attitude, an association with fertility would be a hint of potential selection on political ideology. We therefore investigated on the basis of men and women that have completed, respectively, almost completed reproduction, of three different data sets (the World Value Survey 1981–2014 covering a wide range of countries and developmental levels, *n* = 152,380, the Survey of Health, Ageing and Retirement in Europe of 2005, *n* = 65,912, and the General Social Survey of the United States 1972–2014, *n* ∼ 6200) whether political attitude is associated with number of children. Overall, in the world wide survey, both extreme political attitudes, albeit more pronounced for right/conservative than for left/liberal attitude, are associated with higher average offspring number compared to intermediate attitudes. If countries are analyzed separately, however, the picture is inconsistent, and in most countries, the association is non-significant. In the European and the US-survey, only the political right is associated with above average number of children. The time series of US data from 1972 to 2014 shows that at least in the US-sample, this pattern emerged during the 1990s: in the 1970s and 1980s, also in the US-sample both political extremes had a reproductive advantage, which vanished for left wing individuals during the 1990s. From an evolutionary perspective, we are not able to draw final conclusions as the association between political attitude and reproduction varies across countries and time. Nonetheless, the overall pattern suggests that in human evolutionary history, both left and right political attitudes may have conveyed fitness benefits so that both attitudes have been kept in the population.

## Introduction

There is growing evidence that human ideology, as well as social and political attitudes also have a substantial genetic basis (Hatemi and McDermott, 2012, 2016). First indication that social attitudes have some genetic basis dates back to 1974 (Eaves and Eysenck, 1974) and since then further evidence has accumulated (reviewed in Hatemi and McDermott, 2012). Twin studies (reviewed in Hatemi and McDermott, 2012) with various measures of political attitude and across different cultures found heritability ranging from 0.30 and 0.64. Even though major life history events such as, for instance, job loss, or divorce may modulate the importance of genetic influences on individual attitudes, on longer terms, the proportion of genetic and environmental influences remain rather stable (Hatemi, 2013). Accordingly, political orientation may be among those traits where an evolutionary “interplay” between genes and the environment (Alford et al., 2011; Hatemi and McDermott, 2012) in the sense of a cultural-genetic co-evolution may have happened (Richerson et al., 2010).

In case political attitude indeed has a genetic basis, this basis should either be a product of selection, a by-product of selection for another trait, or both. Such presumptions, however, not only challenge the claims that individual differences of attitudes are solely socially determined but also that humans have been able to “overcome” evolution. Even among evolutionary psychologists the view is widely accepted that at the end of the Pleistocene, human evolution has more or less come to an end (Barkow et al., 1995). The wide use of genetic as well as “big” data, however, has led to novel insights that profoundly challenge the notion that human evolution has come to an end (Stearns et al., 2010; Field et al., 2016).

To our knowledge, this study is the first to examine whether evolution might still be effective in terms of selective scenarios acting on political attitudes also in contemporary populations. This question is based on the assumption that in case of some genetic predisposition of political attitude, an association with fertility would be a hint of potential selection (or by-product selection) on political ideology. Unfortunately, the identification of genes and genomic regions associated with attitudes is challenging, as genome wide association studies on such complex traits usually require a huge number of cases, which is typically only available for biomedical studies. Hence, the so far available data are insufficient to obtain the significances needed to allow drawing reliable conclusions from GWA studies on genotype–phenotype associations (Hatemi and McDermott, 2012; Lockyer et al., 2018). The robust identification of genetic regions associated with “political attitudes” will thus have to wait until sufficiently large data sets will be available. In this study, we therefore confine our analyses on phenotypical data.

In the light that we evolved in groups that provided resources, protection, and security, we hypothesize that in human evolutionary history, both left/liberal attitudes (e.g., being open to change, appreciating new ideas, appreciating the contact with strangers) and right/conservative attitudes (e.g., conserving traditions and culture, being cautious on new developments and strangers) may have provided reproductive advantages. More open individuals may have had benefits by being a source of innovation and fostering contact to strangers, thereby, among others, facilitating access to novel resources and reducing the prevalence of inbreeding (Sikora et al., 2017). More conservative individuals, on the other hand, may have benefitted by the conservation of successful traditions and by being cautious to unpredictable developments and potentially dangerous and violent strangers (Meyer et al., 2015; Curry, 2016). Consequently, we assume that both political attitudes, the so-called “left” and the so-called “right,” may have provided evolutionary advantages, so that both types may have been actively maintained in the population in the sense of a “balancing selection.” Mate preference may be a possible underlying mechanism as it has been shown that people evaluate potential partners more favorably when they have similar political characteristics (Huber and Malhotra, 2017). Under certain conditions such as “times of polarization,” where partisanship gets increasingly important, more extreme political attitudes may provide mating and reproductive advantages, whereas in less polarized situations moderate attitude may convey reproductive benefits. In case the more extremes on both sides have reproductive advantages compared to the more moderate individuals, the frequency of – yet unknown – alleles associated with more extreme political attitude would be expected to increase in a population.

This study aims to analyze the relation between political orientation and reproductive success on a world-wide basis. To test this prediction, we examined whether political attitude provides reproductive advantages. We used three different surveys for our analyses, namely, the World Value Survey (WVS), the Survey of Health, Ageing and Retirement in Europe (SHARE), and the General Social Survey (GSS) of the United States.

## Materials and Methods

### World Value Survey (WVS)

The WVS includes 100 countries world-wide (for list of countries, see Supplementary Table S1), including developed as well as non-developed countries. We analyzed the waves 1981–1984, 1989–1993, 1994–1998, 1999–2004, 2005–2009, and 2010–2014 (number of cases shown in Supplementary Table S2), including a total number of 152,380 individuals (men and women). As the sample includes also countries with a comparably low life expectancy, we included all individuals in the analyses that were older than 40 years at the time of the survey, so that almost all women and most of the men have already completed reproduction, and that also in those countries with low life expectancy, a sufficient number of cases are included. We included the following parameters in the analyses: sex (encoded as 1 = male, 2 = female), number of children [here the precise question is “How many children do you have,” which may comprise some imprecisions; also men not necessarily know their actual number of biological children (this also holds true for the SHARE and GSS data sets)], highest educational attainment (encoded as eight levels, see Supplementary Table S3), scales of income (encoded in 10 steps, surveyed in line with the income distribution of wave and country), the frequency of attendance of religious services (encoded as more than once a week = 6, once a week = 5, special holidays = 4, once a year = 3, less often = 2, never/practically never = 1), age at the time of the survey in years, as well as self-positioning of general political attitude on a 10-item left–right scale (1 = most left, 10 = most right).

In the Supplementary Material, we further analyzed the agreement on a 10-item scale (1 = most left position, 10 = most right position) to the following 10 questions pointing to political attitude: (i) hard work brings success: no…yes; (ii) government should take more responsibility: yes…no; (iii) income should be made more equal vs. we need more income differences; (iv) governmental vs. private ownership; (v) ethnic diversity enriches life vs. erodes life; (vi) homosexuality always justifiable vs. never justifiable; and (vii) abortion always justifiable vs. never justifiable.

We calculated the following linear mixed models: (i) regressing the number of children (on basis of a Poisson error distribution) on self-positioning of general political attitude (respectively the surveyed questions in the Supplementary Material), sex, and education as factors, as well as age, scales of income, and frequency of the attendance of religious services (as continuous numeric variables) with number of the survey wave, and country as random factors; and (ii) including self-positioning of general political attitude (respectively the surveyed questions in the Supplementary Material) as continuous variable in the linear mixed model (a) in terms of a linear term if the relationship between political attitude and number of children is clearly linear (as indicated by a plot), or (b) in terms of a quadratic term (*ax* + *bx*^1^) in the case the relationship between political attitude and number of children is clearly non-linear (i.e., both clearly left- and right-orientated individuals have on average more children than individuals with a moderate attitude). As the countries in the WVS have various economic, social, cultural, and religious backgrounds so that the attitude “left” and “right” may have different meanings, in addition we (i) analyzed the data across WVS waves separately for each country, and (ii) across countries separately for each WVS wave, in each case correcting for sex, age, education, scales of income, and visits of religious services, with either country or wave as random factors (treating individuals as cross-classified among countries and waves).

### Survey of Health, Ageing and Retirement in Europe (SHARE)

We used the 5th wave of the SHARE survey (completed in November 2013^2^; Börsch-Supan, 2018) including 15 European countries as well as Israel, which is the only wave that provides all needed variables. We only analyzed individuals aged older than 45 years (Supplementary Table S4) so that most men and almost all women have already finished reproduction, including a total of 65,912 individuals. We included the following parameters in our analyses: sex (1 = male, 2 = female), number of biological children, political self-positioning on an 11-item scale (0 = most left, 10 = most right), age at the time of the survey in years, percentiles of household income, and highest education (encoded as the seven ISCED 1997 codes^2^). For the SHARE data set, no comparable indicator for the frequency of attendance of religious services is available. We therefore used religious denomination as random factor in the models.

We calculated the following linear mixed models: (i) regressing the number of children (on basis of a Poisson error distribution) on political self-positioning, sex, and education as factors, as well as age and percentiles of income as continuous variables, with country and religious denomination used as random factors (treating individuals as cross-classified among countries and religious denominations), and (ii) including political self-positioning as continuous variable in the linear mixed model in terms of a linear term as the relationship between political self-positioning and number of children in the SHARE survey was clearly linear (as indicated by a plot).

### General Social Survey (GSS) of the United States

We used the GSS of the United States from the years 1972 to 2014, including a total of ∼6200 individuals aged older than 45 years (see Supplementary Table S5). To analyze the time course of the association between political attitude and number of children, we divided this data set into time-intervals 1972–1979, 1980–1989, 1990–1999, 2000–2009, and 2010–2014. We included the following surveyed variables in our analysis: sex (1 = male, 2 = female), number of biological children, political self-positioning (encoded as 1 = extremely liberal, 2 = liberal, 3 = slightly liberal, 4 = moderate, 5 = slightly conservative, 6 = conservative, and 7 = extremely conservative), highest completed education (encoded as: 1 = lower than high school, 2 = high school, 3 = junior college, 4 = bachelor, and 5 = graduate), age at survey in years, income encoded in 12 steps by GSS (see Supplementary Table S6), and the frequency attendance of religious services (encoded as 0 = never, 1 = less once a year, 2 = once a year, 3 = several times a year, 4 = once a month, 5 = 2 × 3 a month, 6 = nearly once a week, 7 = every week, 8 = more than once a week).

We calculated the following general linear mixed models: regressing the number of children (on basis of a Poisson error structure) on political self-positioning, education, and sex as categorical variables, as well as age, frequency attendance of religious services (as numeric variable), and income with year of survey as random factor (i) using the whole data set, (ii) for time intervals 1972–1979, 1980–1989, 1990–1999, 2000–2009, and 2010–2014, and (iii) (in the Supplementary Material) for each survey year separately.

### WVS, SHARE, and GSS

In addition, we calculated the overall variance explained by each linear mixed model and also separately for each explaining variable as well as for the random factors of each linear model, according to Nakagawa and Schielzeth (2013), implemented in the R-library “MuMIn” and the function r.squared GLMM.

## Results

### WVS

**FIGURE 1:**
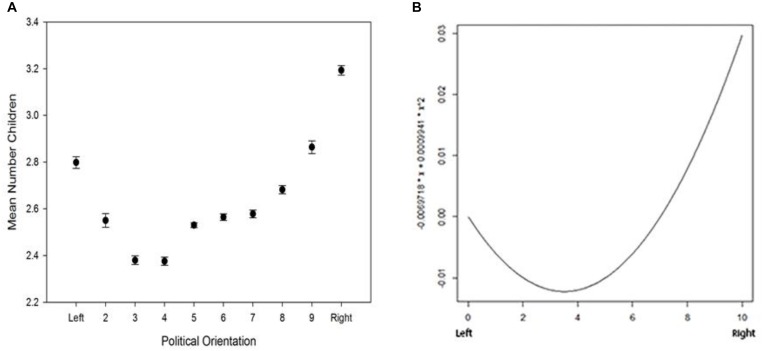
WVS data set: **(A)** political orientation and mean (±SE) number of children and **(B)** quadratic model of political orientation and mean number of children.

In the WVS, overall, we find a u-shaped association between political attitude and number of children: both clearly left and clearly right positioned individuals have, on average, a higher number of children than individuals with an intermediate political attitude. In addition, the highest mean number of children is found in clearly right orientated individuals (Figures 1A,B). This non-linear relationship is confirmed by the association between political attitude (included as quadratic regression) and number of children (Table 1). Age, scales of income, and frequency of attendance of religious services are positively (only significant for age and frequency of attendance of religious services), and increasing education and sex (indicating that in this sample, males have on average more offspring than females) are significantly negatively associated with the number of children. The pattern of a curve linear, quadratic relationship is also confirmed if political attitude is included as a factor (Supplementary Table S7). The quadratic model has a better fit compared to the linear model according to the lower AIC (AIC Linear: 324766, AIC Quadratic: 324468.4); also applying the likelihood ratio test between the linear model and the quadratic model reveals a significant difference (Chi-squared 1 d.f. = 299.6101, *P* < 0.0001).

**Table 1:** WVS: linear mixed model of number of children on basis a Poisson error structure regressing on political orientation included as quadratic term, age, sex, education (lowest education level 1 as reference), scales of income and frequency of attendance of religious services, with wave, and country as random factors.

	Value	SE	*t*-value	*p*-value
(Intercept)	0.6661	0.0342	19.4723	*P* < 0.0001
Age	0.0078	0.0002	40.3882	*P* < 0.0001
Sex female (ref. male)	-0.0238	0.0040	-6.0279	*P* < 0.0001
Self-positioning left–right (linear term)	-0.0076	0.0034	-2.2615	0.0237
Self-positioning left–right (quadratic term)	0.0010	0.0003	3.5621	0.0004
Highest education 2 (ref. 1)	-0.0905	0.0072	-12.6196	*P* < 0.0001
Highest education 3 (ref. 1)	-0.1649	0.0091	-18.0752	*P* < 0.0001
Highest education 4 (ref. 1)	-0.2267	0.0079	-28.6320	*P* < 0.0001
Highest education 5 (ref. 1)	-0.1886	0.0094	-20.0633	*P* < 0.0001
Highest education 6 (ref. 1)	-0.2585	0.0083	-31.1223	*P* < 0.0001
Highest education 7 (ref. 1)	-0.2951	0.0104	-28.3070	*P* < 0.0001
Highest education 8 (ref. 1)	-0.3430	0.0085	-40.5698	*P* < 0.0001
Scales of income encoded in 10 steps	0.0014	0.0010	1.5051	0.1323
Frequency of attendance of religious services	0.0278	0.0012	22.5515	*P* < 0.0001
DF	85,211			
Random factors	Country	Wave	Residuals	
SD:	0.2716306	0.11543	0.90623	

This overall pattern, however, is not consistent for each country surveyed in the WVS. Investigating each country separately, we find that in 10 countries, a significant or marginally significant quadratic relationship of the form -*x* + *x*^2^ indicates that the extremes on both, left and right, are associated with higher average number of children (Table 2A). On the contrary, in Australia, Palestine, Moldovia, and Montenegro, a quadratic relationship of the form *x* -*x*^2^ indicates a reproductive advantage for the political moderate (Table 2B). In 16 countries, we find a significant or marginally significant linear relationship between political orientation and number of children: in eight countries, a positive association indicates a reproductive benefit for the political “left” (Table 3A), and in the other eight countries, a negative association points to a reproductive benefit for the political “right” (Table 3B). The remaining 70 countries of the WVS show no significant association between political orientation and number of children (data not shown).

**Table 2:** Estimates and significances of the linear mixed model of number of children on basis a Poisson error structure regressing on political orientation included as quadratic regression term, age, sex, education (lowest education level 1 as reference), scales of income, and frequency of attendance of religious services, with wave as random factor, separately for single countries of the WVS: **(A)** extreme political attitude is associated with reproductive advantages and **(B)** political moderate attitude is associated with reproductive advantages.

	*X*	*P*	*x*^2^	*P*	*R*^2^
**(A)**					
Azerbaijan	-0.0719286	^∗∗∗^	0.0062184	^∗∗^	0.01701
Chile	-0.0634107	^∗^	0.0050126	^∗^	0.00155
China	-0.0634107	^∗^	0.0050126	^∗^	0.00140
Mali	-0.1295782	.	0.0094600	.	0.01311
Mexico	-0.0331836	.	0.0027538	.	0.01234
Russia	-0.0674964	^∗∗^	0.0060292	^∗∗^	0.00307
Ukraine	-0.0414779	.	0.0032432	.	0.00125
Great Britain	-0.1615588	^∗^	0.0108873	.	0.01386
Tanzania	-0.0883949	.	0.0070985	.	0.10076
Zambia	-0.1144353	.	0.0093888	.	0.03030
**(B)**					
Australia	0.08669884	^∗∗∗∗^	-0.00627913	^∗∗^	0.00880
Palestine	0.1309962	^∗^	-0.0108071	^∗^	0.03275
Moldova	0.07708511	^∗∗^	-0.00666613	.	0.00731
Montenegro	0.0759482	.	-0.0075943	.	0.00236

*R^2^ estimates for mixed models according to Nakagawa and Schielzeth (2013). ^∗^p < 0.05, ^∗∗^p < 0.01, ^∗∗∗^p < 0.001.*

**Table 3:** Estimates and significances of the linear mixed model of number of children on basis a Poisson error structure regressing on political orientation included as a linear term, age, sex, education (lowest education level 1 as reference), scales of income, and frequency of attendance of religious services, with wave as random factor, separately for single countries of the WVS: **(A)** political “left” is associated with reproductive advantages and **(B)** political “right” is associated with reproductive advantages.

Country	*X*	*P*	*R*^2^
**(A)**			
Czech Rep.	-0.0304534	^∗∗∗^	0.00745
Bahrain	-0.0303582	^∗^	0.00250
Puerto Rico	-0.0210094	^∗^	0.00206
Libya	-0.0206035	^∗^	0.00528
Norway	-0.0189647	^∗^	0.00059
Poland	-0.0152985	^∗^	0.00014
Peru	-0.0129464	^∗^	0.00258
Sweden	-0.0105985	.	0.00005
**(B)**			
Egypt	0.0131942	.	0.00010
Turkey	0.0172853	^∗∗∗^	0.04125
Kyrgyzstan	0.0176033	^∗∗^	0.00687
Indonesia	0.0181714	^∗^	0.01177
Rwanda	0.0213168	^∗^	0.00162
Macedonia	0.0222626	^∗∗^	0.00783
Iran	0.0268428	^∗^	0.00824
Ethiopia	0.106196	^∗∗^	0.00004

*R^2^ estimates for mixed models according to Nakagawa and Schielzeth (2013). ^∗^p < 0.05, ^∗∗^p < 0.01, ^∗∗∗^p < 0.001.*

**FIGURE 2:**
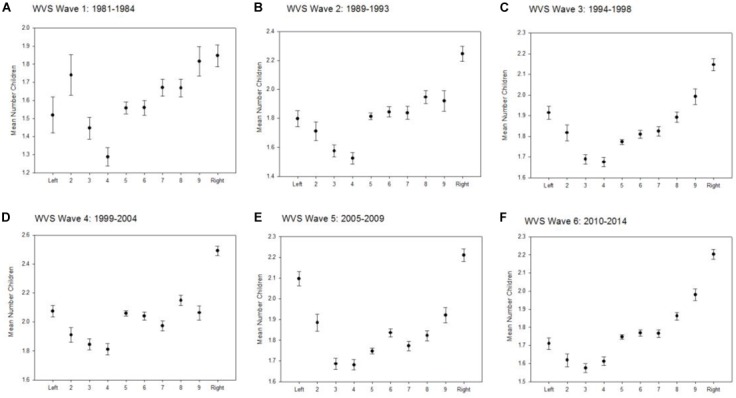
**(A–F)** Political attitude and mean (±SE) number of children separately for each WVS wave.

By including all countries of the WVS but analyzing each wave separately, all plots of political attitude vs. average number of children suggest a reproductive advantage for the extremes, both left and right (Figures 2A–F). The models are less clear: only in wave 5, we find a reproductive advantage for both left and right, whereas in three waves, a reproductive advantage is only found for the political right, and in two waves, the association remains non-significant (Table 4).

**Table 4:** Estimates and significances of the linear mixed model of number of children on basis a Poisson error structure regressing on political orientation, age, sex, education (lowest education level 1 as reference), scales of income, and frequency of attendance of religious services, with country as random factor, separately for single waves of the WVS.

	*X*	*P*	*x*^2^	*P*
WAVE 1 1981–1984	0.0160872	<0.05		
WAVE2 1990–1994	-0.0178059	ns	0.0018924	ns
WAVE 3 1995–1998	0.0054185	<0.05		
WAVE 4 1999–2004	0.0052286	ns		
WAVE 5 2005–2009	-0.016012	<0.05	0.0018141	<0.05
WAVE 6 2010–2014	0.0063071	<0.001		

*ns: not significant*

Overall, 19.1 % of the variance is explained by random factors country and wave and about 8% by the fixed factors (education: 3.98%, age: 2.53%, income: 0.68%, frequency of attendance of religious services: 0.65%, political orientation: 0.15%, sex: 0.0099%). Although political orientation only explains a small proportion of the variance in the overall model, in the single country analysis (significant associations only), the variance explained ranges widely from 0.0045% (Ethopia) up to 10.1% (Tanzania).

Overall, for the surveyed questions on a 10-item scale-agreement to the statements, “hard work brings success: no…yes,” “government should take more responsibility: yes…no,” ”income should be made more equal vs. we need more income differences,” and “governmental vs. private ownership,” we also find a significant quadratic association, with higher average number of children in individuals with the most extreme compared to intermediate positions (Supplementary Figures S1–S4 and Supplementary Tables S8–S11). For the question on “ethnic diversity enriches life vs. erodes life,” the plot of the raw data also suggests a quadratic association but in the general linear mixed model, the association remained non-significant (Supplementary Figure S5 and Supplementary Table S12). For the survey questions on “homosexuality always justifiable vs. never justifiable,” and “abortion always justifiable vs. never justifiable,” a clear linear relationship is seen, with rejection of homosexuality and abortion, respectively, being increasingly associated with higher number of children (Supplementary Figures S6, S7 and Supplementary Tables S13, S14).

### SHARE

**FIGURE 3:**
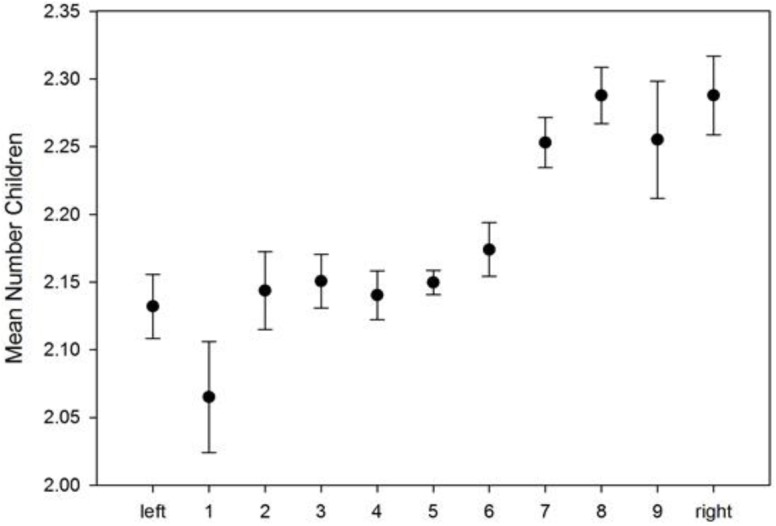
SHARE data: political attitude and mean (±SE) number of children.

In the European countries plus Israel included in SHARE, right-wing individuals have, on average, more children than intermediate or left-wing individuals (Figure 3). This finding is supported by a significant positive linear association between political orientation and number of children, corrected for sex, age, education, income, with country used as random factor (Table 5), as well as by the linear mixed model of number of children regressing on political orientation as factor (Supplementary Table S15). The latter model indicates that individuals with the three most conservative (i.e., right-wing) attitudes have significantly more children compared to all other groups. In addition, in both models, age and household income percentiles are significantly positively, whereas education is significantly negatively associated with the number of children (Table 5 and Supplementary Table S15). We also tested a curve-linear relationship, which remained non-significant (data not shown). The overall model in the SHARE data set explains 5.2%, all fixed factors explain 1.7% (political attitude 0.05%, education 1.5%, age 0.16%, household income: 0.0059%, sex: 0.0021%) and the random factor country explains 3.51% of the variance.

**Table 5:** SHARE: linear mixed model of number of children on basis a Poisson error structure regressing on political orientation included as linear term, age, sex, education, and scales of income, with country as random factor.

	Value	SE	*t*-value	*p*-value
(Intercept)	0.8011	0.0405	19.769	*p* < 0.0001
Age	0.0015	0.0003	5.448	*p* < 0.0001
Sex female (ref. male)	-0.0018	0.0052	-0.347	0.7286
Self-positioning left–right (linear term)	0.0055	0.0011	4.743	*p* < 0.0001
Highest education 2 (ref. 1)	-0.0896	0.0143	-6.279	*p* < 0.0001
Highest education 3 (ref. 1)	-0.2023	0.0147	-13.752	*p* < 0.0001
Highest education 4 (ref. 1)	-0.2542	0.0145	-17.508	*p* < 0.0001
Highest education 5 (ref. 1)	-0.2903	0.0189	-15.380	*p* < 0.0001
Highest education 6 (ref. 1)	-0.2712	0.0149	-18.168	*p* < 0.0001
Highest education 7 (ref. 1)	-0.2628	0.0299	-8.779	*p* < 0.0001
Household income percentiles	0.0115	0.0010	11.715	*p* < 0.0001
DF	55,224			
Random factors	Intercept country	Residuals		
SD:	0.1201166	0.8900663		

### GSS

**FIGURE 4:**
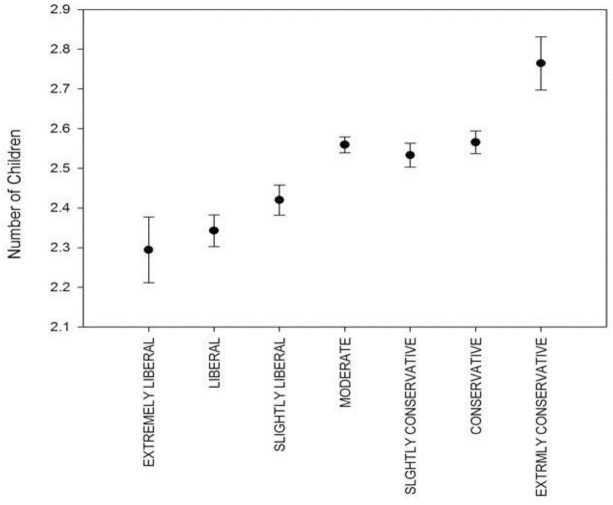
GSS-data: political orientation and mean (±SE) number of children.

In the GSS, overall (i.e., analyzing the complete data set) average number of children increases with increasingly conservative attitude (Figure 4). This finding is confirmed by the linear mixed model (Table 6), where signs and significances indicate that the number of children is higher in increasingly conservative as compared to extremely liberal individuals. Estimates and significances do not change significantly if race (white, afro-American and other) is included in the overall model (data not shown). We also tested a curve-linear relationship, which remained non-significant (data not shown).

**Table 6:** GSS: linear mixed model of number of children on basis a Poisson error structure regressing on political orientation included as factor (most liberal as reference), age, sex, education (lowest education level “lower than high school” as reference), income, and frequency of attendance of religious services, with year of survey as random factor.

	Value	SE	*t*-value	*p*-value
(Intercept)	0.91610	0.07188	12.74492	*P* < 0.0001
Age	0.00299	0.00089	3.36146	0.00080
Sex female (ref. male)	-0.03603	0.01432	-2.51668	0.01190
Liberal (ref.: extremely liberal)	0.0974634	0.05002297	1.948372	0.0514
Slightly liberal (ref.: extremely liberal)	0.1132255	0.04975543	2.275641	0.0229
Moderate (ref.: extremely liberal)	0.154331	0.04671412	3.303733	0.001
Slightly conservative (ref.: extremely liberal)	0.1554391	0.04821662	3.223766	0.0013
Conservative (ref.: extremely liberal)	0.1609268	0.04840244	3.324766	0.0009
Extremely conservative (ref.: extremely liberal)	0.1824526	0.05640799	3.234517	0.0012
High school (ref.: Lt high school)	-0.17865	0.01782	-10.02615	*P* < 0.0001
Junior college (ref.: Lt high school)	-0.25791	0.03492	-7.38510	*P* < 0.0001
Bachelor (ref.: Lt high school)	-0.37715	0.02488	-15.15600	*P* < 0.0001
Graduate (ref.: Lt high school)	-0.39310	0.02742	-14.33621	*P* < 0.0001
Income	-0.00406	0.00242	-1.67659	0.09370
Frequency of attendance of religious services	0.02689	0.00256	10.51502	*P* < 0.0001
*N*	31,244			
Random survey year	(Intercept)	Residual		
SD:	0.07580	1.10977		

**FIGURE 5:**
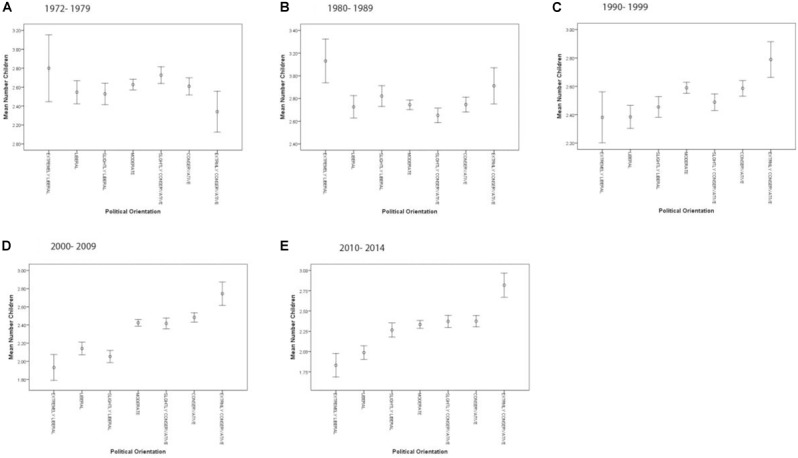
**(A–E)** GSS: political orientation and mean number of children aggregated on survey decades.

Analyzing time intervals separately reveals that the reproductive advantage for conservatives found in the overall data set is only present in the more recent intervals (i.e., 1990–1999, 2000–2009, 2010–2014; Figures 5C–E), whereas prior to the 1990s (i.e., 1972–1979 and 1980–1989), the pattern is more blurred, with highest mean number of children found in the most liberal individuals (Figures 5A,B). The linear mixed models for each time interval are shown in Supplementary Tables S16–S20.

Also by analyzing each year separately, the reproductive advantage for the political right gets increasingly more straightforward in the later years (Supplementary Figure S8), although owing to smaller sample sizes, only few estimates remain significant (Supplementary Table S21); 9.33% of the overall variance is explained by the overall model (0.38% random factor survey year), and 8.95% is explained by the fixed factors (0.4% by the political orientation, education 5.87%, frequency of attendance of religious services: 1.58%, income: 0.95%, age: 0.38%, and sex: 0.17%).

## Discussion

Overall, in the worldwide sample (WVS), we find a reproductive advantage for the more extreme political positions, both “right” and “left.” In addition, overall right attitude is associated with higher average number of children than overall left attitude. However, the pattern for the single countries is inconsistent: in most countries, we find no significant association between political attitude and number of children at all, though this may be caused by low sample size, which seems also to hold true for the analysis of single waves. In addition, in those countries, where a significant association is found, the direction of association does not reflect a consistent pattern: in some countries, we find a reproductive advantage either for the political “left” or for the political “right,” in other countries, we find a reproductive advantage for the moderate. Also, overall political orientation only explains a very small proportion of the variance in reproduction, although the proportion strongly varies from country to country from negligible 0.0045% for Ethiopia, 4% in Turkey, up to reasonable 10% in Tanzania. We can only speculate on the reasons for these patterns. Possibly, the association is influenced by economic development (e.g., GDP, GINI, or other indicators) or political system, which may be question for future research. From an evolutionary perspective, although the overall WVS pattern of a reproductive advantage for both, more extreme left wing and more extreme right wing individuals, may suggest balancing selection (Relethford, 2012), where both phenotypes are advantageous and thus kept in the population, this view cannot be inferred from the analysis of single countries. Maybe different selection scenarios act differently across time and space, leading to the overall pattern of a balancing selection. This conclusion is supported by some but not all other items sampled by WVS, particularly the questions on economic attitude that also show both a left- and a right-wing reproductive advantage. To come to a more robust conclusion, additional data are needed, for instance, from more egalitarian societies.

In contrast to the WVS, in the recent European sample (including also Israel) (SHARE), we find no reproductive benefit at all for extreme left positions. On the contrary, the average number of children increases with increasingly conservative political attitude. The same association holds true for the US-sample (GSS), albeit only if analyzing the overall data set. Differences in average offspring number are less pronounced in the GSS (comprising data from 1974 to 2014), however, than in the SHARE (a cross-sectional survey completed in year 2013). We assume that the reason for this discrepancy lies in the finding that the association between political attitude and offspring number in the GSS changed with time: although a reproductive advantage for more conservative individuals arises in time intervals 1990–1999, 2000–2009, 2010–2014, prior to 1990, the highest number of children is found among the most liberal individuals.

This shifting association in the GSS may be explained by the negative effect of higher education on offspring number, primarily via postponing of reproduction (Ní Bhrolcháin and Beaujouan, 2012) as progression to graduate education is more pronounced in liberals than in conservatives. However, an originally significant interaction of political attitude and education on number of children (data not shown) disappeared as soon as the frequency of religious attendance was included in the model, indicating a more complex interrelation among religiousness, education, political attitude, and reproduction. The positive interactions between the attendance of religious services and education (Supplementary Table S22) suggests that particularly in better educated individuals, religiousness is associated with a higher number of children.

Studies show that political attitude has a genetic basis: the first study exploring genetic influences on individual differences in political attitude was published by Eaves and Eysenck (1974) who used a twin study to estimated genetic and environmental sources of variance. They found that monozygotic twins correlated more highly than dizygotic (DZ) co-twins on measures of ideology on a constructed scale of attitudes including, among others, questions on death penalty, ethnocentrism, morality, unions, un-employment, and abortion, with a relative amount of variance due to additive genetic influences between 0.54 and 0.65. Additionally, parent’s and children’s resemblance in political attitudes seem to be more a function of genetic transformation and individual history than social learning (Hatemi and McDermott, 2012). Nonetheless, as available data are insufficient for reliable GWA studies on genotype–phenotype associations (Hatemi and McDermott, 2012; Lockyer et al., 2018), a search of traces of selection on yet uncertain genomic loci makes less sense. We do not know with sufficient reproducibility, whether and which genetic loci are associated with political orientation. Thus, at the moment interpretations of our findings must remain purely speculative.

However, based on the very recent finding that polygenic selection may act within previously unpredicted short time intervals (Field et al., 2016), we assume that the scenarios of selection acting in short time intervals are not implausible, as such scenarios have been demonstrated for other phenotypes such as the human pelvis (Mitteroecker and Fischer, 2016), educational attainment (Beauchamp, 2016), or age at first birth and body mass related to reproduction (Sanjak et al., 2017).

From our results, we can merely speculate that alleles associated with either side of the political spectrum might spread in a population (this holds true for the case of balancing as well as diversifying selection). In addition, the changing pattern with time in the US-sample (GSS) might point to a change from balancing selection to a directional selection in an ongoing process of cultural and genetic co-evolution (Richerson et al., 2010). It appears that in the US-sample, possibly due to more pronounced education and an interaction with religiousness, reproductive patterns may have shifted toward the right wing.

In the WVS, we further find that sex is negatively associated with the number of children. Again, we could only speculate on possible reasons for this curious result. As expected, education is significantly negatively and the frequency of the attendance of religious service is significantly positively associated with the number of children. Also a higher age of the respondents predicts higher number of children, indicating that after the age of 40, most but not all individuals have completed reproduction. The association between income and number of children is non-significantly positive. An analysis separately by sex (data not shown) shows that, in accordance with the literature (Fieder et al., 2005; Hopcroft, 2006, 2015; Fieder and Huber, 2007; Nettle and Pollet, 2008), the effect is only positive in men but not women.

On basis of our data, we are not able to draw any final conclusion. But we suggest that although the association between political attitude and reproduction varies across countries and time, the overall pattern indicates that at least in pre-western societies, both a more liberal and a more conservative attitude may have conveyed evolutionary benefits in terms of higher reproduction, so that both attitudes have been kept in the gene pool and may still be influential in modern societies, even though liberal attitudes no longer comprise reproductive advantages.

### Limitations of the Study

Particularly in the case of WVS, we cannot be sure that in such a diverse worldwide sample of countries, all the participants have the same understanding of “left or right.” Number of children differs between men and women, a problem that should not occur in a representative data set. However, as the data have been surveyed by the “WVS organization” we are not able to overcome this limitation.

## Author Contributions

MF analyzed the data and wrote the article. SH wrote the article.

## Conflict of Interest Statement

The authors declare that the research was conducted in the absence of any commercial or financial relationships that could be construed as a potential conflict of interest.

## Footnotes

1 http://ec.europa.eu/eurostat/statistics-explained/index.php?title=International_Standard_Classification_of_Education_(ISCED)

2 http://www.share-project.org/data-documentation/waves-overview/wave-5.html
