# Supplementary material for: Political Attitude and Fertility: Is There a Selection for the Political Extreme?
Source: Front Psychol. 2018 Nov 27;9:2343. doi: 10.3389/fpsyg.2018.02343 (PMC6277747; doi:10.3389/fpsyg.2018.02343)
Supplement: Supplementary file 1 [file Table_1.DOCX]

**Supplementary Material**

**Table S1)** World Value Survey: Countries and number of cases

|  | | N | % |
| --- | --- | --- | --- |
| Country | Albania | 947 | .6 |
|  | Algeria | 856 | .6 |
|  | Andorra | 460 | .3 |
|  | Azerbaijan | 1189 | .8 |
|  | Argentina | 3100 | 2.0 |
|  | Australia | 3597 | 2.4 |
|  | Bahrain | 576 | .4 |
|  | Bangladesh | 715 | .5 |
|  | Armenia | 1447 | .9 |
|  | Bosnia | 564 | .4 |
|  | Brazil | 2091 | 1.4 |
|  | Bulgaria | 1245 | .8 |
|  | Belarus | 2354 | 1.5 |
|  | Canada | 2517 | 1.7 |
|  | Chile | 2614 | 1.7 |
|  | China | 3997 | 2.6 |
|  | Taiwan | 1751 | 1.1 |
|  | Colombia | 3701 | 2.4 |
|  | Croatia | 616 | .4 |
|  | Cyprus | 1012 | .7 |
|  | Czech Rep. | 1219 | .8 |
|  | Dominican Rep. | 58 | .0 |
|  | Ecuador | 515 | .3 |
|  | El Salvador | 470 | .3 |
|  | Ethiopia | 203 | .1 |
|  | Estonia | 1529 | 1.0 |
|  | Finland | 1089 | .7 |
|  | France | 582 | .4 |
|  | Georgia | 1783 | 1.2 |
|  | Palestine | 342 | .2 |
|  | Germany | 3834 | 2.5 |
|  | Ghana | 703 | .5 |
|  | Guatemala | 283 | .2 |
|  | Hong Kong | 1295 | .8 |
|  | Hungary | 934 | .6 |
|  | India | 3725 | 2.4 |
|  | Indonesia | 1404 | .9 |
|  | Iran | 1360 | .9 |
|  | Iraq | 2161 | 1.4 |
|  | Israel | 558 | .4 |
|  | Italy | 588 | .4 |
|  | Japan | 4343 | 2.9 |
|  | Kazakhstan | 651 | .4 |
|  | Jordan | 1316 | .9 |
|  | South Korea | 2721 | 1.8 |
|  | Kuwait | 377 | .2 |
|  | Kyrgyzstan | 1006 | .7 |
|  | Lebanon | 516 | .3 |
|  | Latvia | 608 | .4 |
|  | Libya | 897 | .6 |
|  | Lithuania | 522 | .3 |
|  | Malaysia | 967 | .6 |
|  | Mali | 482 | .3 |
|  | Mexico | 3482 | 2.3 |
|  | Moldova | 1607 | 1.1 |
|  | Morocco | 1142 | .7 |
|  | Netherlands | 2019 | 1.3 |
|  | New Zealand | 1939 | 1.3 |
|  | Nigeria | 1200 | .8 |
|  | Norway | 1187 | .8 |
|  | Pakistan | 1232 | .8 |
|  | Peru | 1970 | 1.3 |
|  | Philippines | 1497 | 1.0 |
|  | Poland | 2431 | 1.6 |
|  | Puerto Rico | 848 | .6 |
|  | Qatar | 423 | .3 |
|  | Romania | 2699 | 1.8 |
|  | Russia | 4603 | 3.0 |
|  | Rwanda | 746 | .5 |
|  | Saudi Arabia | 329 | .2 |
|  | Singapore | 1416 | .9 |
|  | Slovakia | 849 | .6 |
|  | Viet Nam | 1193 | .8 |
|  | Slovenia | 1846 | 1.2 |
|  | South Africa | 6387 | 4.2 |
|  | Zimbabwe | 673 | .4 |
|  | Spain | 3429 | 2.3 |
|  | Sweden | 3059 | 2.0 |
|  | Switzerland | 2371 | 1.6 |
|  | Thailand | 1657 | 1.1 |
|  | Trinidad and Tobago | 1068 | .7 |
|  | Tunisia | 489 | .3 |
|  | Turkey | 3307 | 2.2 |
|  | Uganda | 133 | .1 |
|  | Ukraine | 3015 | 2.0 |
|  | Macedonia | 962 | .6 |
|  | Egypt | 3185 | 2.1 |
|  | Great Britain | 1167 | .8 |
|  | Tanzania | 449 | .3 |
|  | United States | 4758 | 3.1 |
|  | Burkina Faso | 404 | .3 |
|  | Uruguay | 1716 | 1.1 |
|  | Uzbekistan | 612 | .4 |
|  | Venezuela | 822 | .5 |
|  | Yemen | 264 | .2 |
|  | Serbia and Montenegro | 627 | .4 |
|  | Zambia | 241 | .2 |
|  | Serbia | 1450 | 1.0 |
|  | Montenegro | 715 | .5 |
|  | Bosnia | 372 | .2 |
|  | Total | 152380 | 100.0 |

**Table S2)** World Value Survey Number of cases for each wave

|  | | N | % |
| --- | --- | --- | --- |
| Wave | 1981-1984 | 3866 | 2.5 |
|  | 1989-1993 | 10106 | 6.6 |
|  | 1994-1998 | 34032 | 22.3 |
|  | 1999-2004 | 23777 | 15.6 |
|  | 2005-2009 | 39335 | 25.8 |
|  | 2010-2014 | 41264 | 27.1 |
|  | Total | 152380 | 100.0 |

**Table S3)** Education levels of the World Value Survey participants

|  | | **N** | **%** |
| --- | --- | --- | --- |
| Education | Inadequately completed elementary education | 15059 | 11.56% |
|  | Completed (compulsory) elementary education | 25444 | 19.53% |
|  | Incomplete secondary school: technical/vocational type/(Comp | 10242 | 7.86% |
|  | Complete secondary school: technical/vocational type/Seconda | 23083 | 17.72% |
|  | Incomplete secondary: university-preparatory type/Secondary, | 9902 | 7.60% |
|  | Complete secondary: university-preparatory type/Full seconda | 19412 | 14.90% |
|  | Some university without degree/Higher education - lower-leve | 7547 | 5.79% |
|  | University with degree/Higher education - upper-level tertia | 19580 | 15.03% |

**Table S4)** Countries and number of cases included in the SHARE survey, wave 5.

|  | | N | % |
| --- | --- | --- | --- |
| Country | Austria | 4356 | 6.6 |
|  | Germany | 5711 | 8.7 |
|  | Sweden | 4542 | 6.9 |
|  | Netherlands | 4154 | 6.3 |
|  | Spain | 6667 | 10.1 |
|  | Italy | 4725 | 7.2 |
|  | France | 4486 | 6.8 |
|  | Denmark | 4129 | 6.3 |
|  | Switzerland | 3037 | 4.6 |
|  | Belgium | 5613 | 8.5 |
|  | Israel | 2593 | 3.9 |
|  | Czech Republic | 5606 | 8.5 |
|  | Luxembourg | 1605 | 2.4 |
|  | Slovenia | 2954 | 4.5 |
|  | Estonia | 5734 | 8.7 |
|  | Total | 65912 |  |

**Table S5)** Number of cases for each wave in the GSS

| **Year** | **Number Cases** | **%** |
| --- | --- | --- |
| 1972 | 768 | 2.8 |
| 1973 | 684 | 2.5 |
| 1974 | 665 | 2.4 |
| 1975 | 657 | 2.4 |
| 1976 | 688 | 2.5 |
| 1977 | 691 | 2.5 |
| 1978 | 639 | 2.3 |
| 1980 | 656 | 2.4 |
| 1982 | 823 | 3.0 |
| 1983 | 666 | 2.4 |
| 1984 | 598 | 2.2 |
| 1985 | 698 | 2.5 |
| 1986 | 626 | 2.3 |
| 1987 | 760 | 2.8 |
| 1988 | 610 | 2.2 |
| 1989 | 673 | 2.5 |
| 1990 | 586 | 2.1 |
| 1991 | 638 | 2.3 |
| 1993 | 706 | 2.6 |
| 1994 | 1341 | 4.9 |
| 1996 | 1232 | 4.5 |
| 1998 | 1226 | 4.5 |
| 2000 | 1258 | 4.6 |
| 2002 | 1312 | 4.8 |
| 2004 | 1340 | 4.9 |
| 2006 | 2274 | 8.3 |
| 2008 | 1069 | 3.9 |
| 2010 | 1076 | 3.9 |
| 2012 | 1043 | 3.8 |
| 2014 | 1385 | 5.1 |

**Table S6)** Income levels for the GSS

|  | N | % |
| --- | --- | --- |
| LT $1000 | 302 | 3.43% |
| $1000 TO 2999 | 430 | 4.88% |
| $3000 TO 3999 | 305 | 3.46% |
| $4000 TO 4999 | 267 | 3.03% |
| $5000 TO 5999 | 247 | 2.80% |
| $6000 TO 6999 | 277 | 3.14% |
| $7000 TO 7999 | 230 | 2.61% |
| $8000 TO 9999 | 447 | 5.07% |
| $10000 - 14999 | 1254 | 14.23% |
| $15000 - 19999 | 922 | 10.46% |
| $20000 - 24999 | 942 | 10.69% |
| $25000 OR MORE | 3190 | 36.20% |

**Table S7)** WVS: Linear mixed model with number of children regressing on political orientation included as factor (most right as reference), age, sex, education (lowest education level 1 as reference), and scales of income and the frequency of attendance of religious services, on basis a Poisson error structure, with wave, and country as random factors.

|  | **Value** | **Std.Error** | **t-value** | **p-value** |
| --- | --- | --- | --- | --- |
| (Intercept) | 0.6936399 | 0.033566 | 20.66497 | P < 0.0001 |
| Age | 0.00783 | 0.00019 | 40.39337 | P < 0.0001 |
| Sex female (ref. male) | -0.02399 | 0.00396 | -6.06265 | P < 0.0001 |
| Self Positioning left 1 (ref. right 10) | -0.03263 | 0.01013 | -3.22127 | 0.0013 |
| Self Positioning 2 (ref. right 10) | -0.04336 | 0.01229 | -3.52660 | 0.0004 |
| Self Positioning 3 (ref. right 10) | -0.04762 | 0.00994 | -4.79262 | P < 0.0001 |
| Self Positioning 4 (ref. right 10) | -0.04801 | 0.00978 | -4.90929 | P < 0.0001 |
| Self Positioning 5 (ref. right 10) | -0.03488 | 0.00722 | -4.83157 | P < 0.0001 |
| Self Positioning 6 (ref. right 10) | -0.04171 | 0.00810 | -5.14980 | P < 0.0001 |
| Self Positioning 7 (ref. right 10) | -0.03488 | 0.00878 | -3.97106 | 0.0001 |
| Self Positioning 8 (ref. right 10) | -0.02750 | 0.00863 | -3.18813 | 0.0014 |
| Self Positioning 9 (ref. right 10) | -0.02203 | 0.01060 | -2.07746 | 0.0378 |
| Highest Education 2 (ref. 1) | -0.09059 | 0.00717 | -12.63397 | P < 0.0001 |
| Highest Education 3 (ref. 1) | -0.16513 | 0.00913 | -18.09352 | P < 0.0001 |
| Highest Education 4 (ref. 1) | -0.22682 | 0.00792 | -28.64774 | P < 0.0001 |
| Highest Education 5 (ref. 1) | -0.18870 | 0.00940 | -20.07092 | P < 0.0001 |
| Highest Education 6 (ref. 1) | -0.25858 | 0.00831 | -31.13270 | P < 0.0001 |
| Highest Education 7 (ref. 1) | -0.29494 | 0.01043 | -28.29133 | P < 0.0001 |
| Highest Education 8 (ref. 1) | -0.34274 | 0.00845 | -40.53827 | P < 0.0001 |
| Scales encoded in 10 steps | 0.00156 | 0.00096 | 1.62603 | 0.1039 |
| frequency of attendance of religious services | 0.02783 | 0.00123 | 22.53804 | P < 0.0001 |
| DF | 84224 |  |  |  |
| Random factors | Intercept Country | Intercept Wave | Residuals |  |
| StdDev: | 0.2875677 | 0.103237 | 0.9010263 |  |


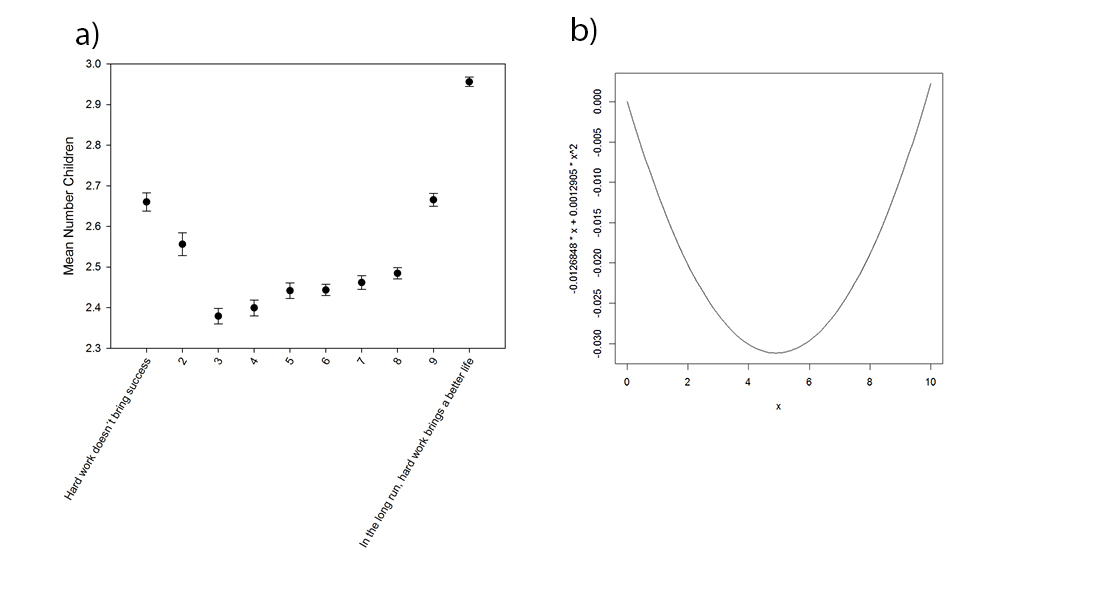


**Figure S1)** WVS data set: attitude towards “hard work” a) mean number of children, standard errors; b) quadratic model.

**Table S8)** WVS: Linear mixed model “hard- work”

|  | **Value** | **Std.Error** | **t-value** | **p-value** |
| --- | --- | --- | --- | --- |
| (Intercept) | 0.6733 | 0.0339 | 19.8533 | 0 |
| Age | 0.0073 | 0.0002 | 37.8856 | 0 |
| Sex female (ref. male) | -0.0138 | 0.0039 | -3.4945 | 0.0005 |
| Hard Work brings no success - brings success (linear term) | -0.0127 | 0.0033 | -3.7955 | 0.0001 |
| Hard Work brings no success - brings success (quadratic term) | 0.0013 | 0.0003 | 4.7528 | 0 |
| Highest Education 2 (ref. 1) | -0.0810 | 0.0071 | -11.4687 | 0 |
| Highest Education 3 (ref. 1) | -0.1514 | 0.0090 | -16.8647 | 0 |
| Highest Education 4 (ref. 1) | -0.2175 | 0.0077 | -28.0735 | 0 |
| Highest Education 5 (ref. 1) | -0.1789 | 0.0095 | -18.8004 | 0 |
| Highest Education 6 (ref. 1) | -0.2430 | 0.0083 | -29.3080 | 0 |
| Highest Education 7 (ref. 1) | -0.2824 | 0.0107 | -26.3685 | 0 |
| Highest Education 8 (ref. 1) | -0.3229 | 0.0085 | -38.1140 | 0 |
| Scales encoded in 10 steps | 0.0017 | 0.0010 | 1.7442 | 0.0811 |
| frequency of attendance of religious services | 0.026102 | 0.001228 | 21.26268 | 0 |


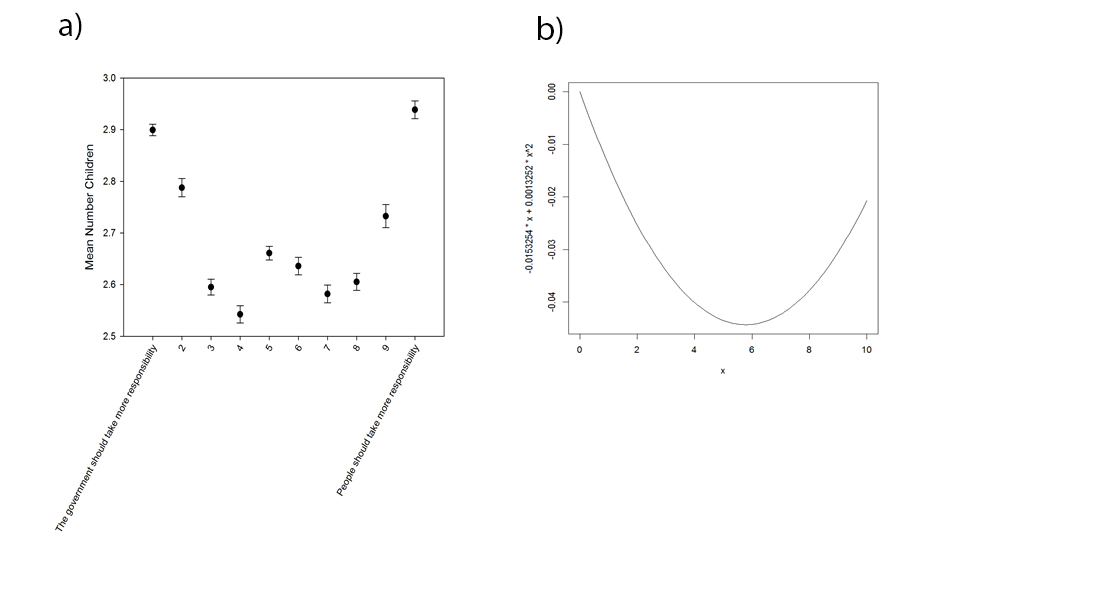


**Figure S2)** WVS data set: attitude towards “governmental responsibility” a) mean number of children, standard errors; b) quadratic model.

**Table S9)** WVS: Linear mixed model “governmental responsibility”

|  | **Value** | **Std.Error** | **t-value** | **p-value** |
| --- | --- | --- | --- | --- |
| (Intercept) | 0.7088 | 0.0326 | 21.7264 | 0 |
| Age | 0.0076 | 0.0002 | 44.2160 | 0 |
| Sex female (ref. male) | -0.0191 | 0.0035 | -5.4554 | 0 |
| gonverment morfe responsible - less responsible (linear term) | -0.0153 | 0.0024 | -6.3567 | 0 |
| gonverment morfe responsible - less responsible (quadratic term) | 0.0013 | 0.0002 | 5.8829 | 0 |
| Highest Education 2 (ref. 1) | -0.0815 | 0.0061 | -13.3430 | 0 |
| Highest Education 3 (ref. 1) | -0.1587 | 0.0080 | -19.9124 | 0 |
| Highest Education 4 (ref. 1) | -0.2192 | 0.0068 | -32.1206 | 0 |
| Highest Education 5 (ref. 1) | -0.1858 | 0.0083 | -22.5122 | 0 |
| Highest Education 6 (ref. 1) | -0.2516 | 0.0072 | -34.7683 | 0 |
| Highest Education 7 (ref. 1) | -0.2906 | 0.0094 | -30.9336 | 0 |
| Highest Education 8 (ref. 1) | -0.3396 | 0.0074 | -45.7106 | 0 |
| Scales encoded in 10 steps | 0.0015 | 0.0009 | 1.6921 | 0.0906 |
| frequency of attendance of religious services | 0.025376 | 0.00108 | 23.49454 | 0 |


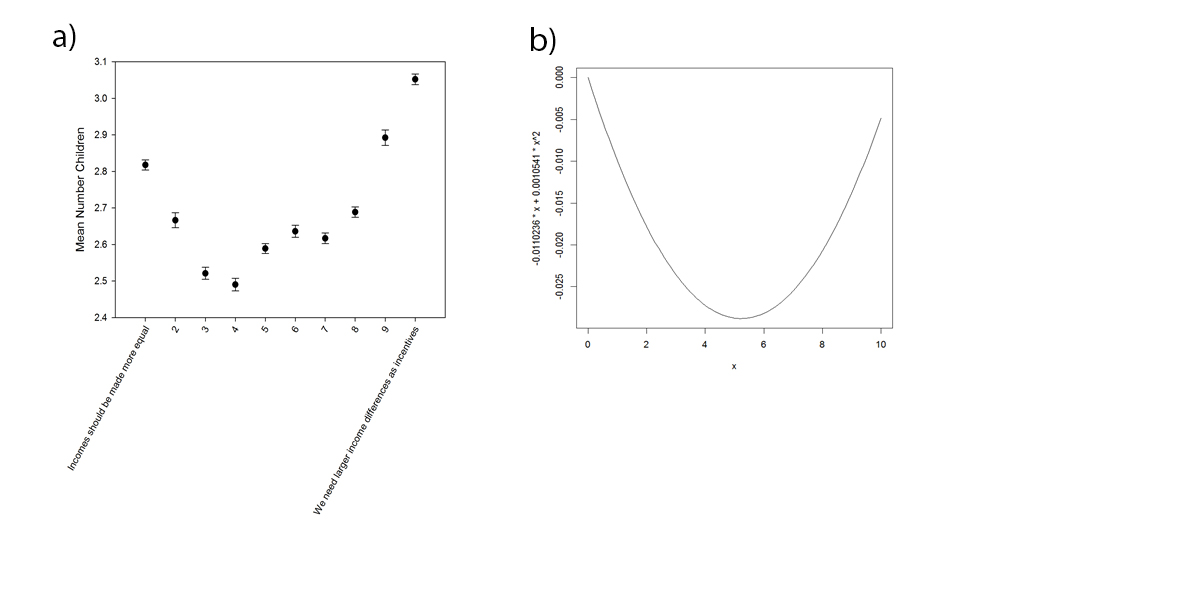


**Figure S3)** WVS data set: attitude towards “income differences” a) mean number of children, standard errors; b) quadratic model.

**Table S10)** WVS: Linear mixed model “income- differences”

|  | **Value** | **Std.Error** | **t-value** | **p-value** |
| --- | --- | --- | --- | --- |
| (Intercept) | 0.6978 | 0.0328 | 21.2444 | 0 |
| Age | 0.0077 | 0.0002 | 44.0787 | 0 |
| Sex female (ref. male) | -0.0207 | 0.0035 | -5.8531 | 0 |
| income more equal - more differences (linear term) | -0.0110 | 0.0025 | -4.3244 | 0 |
| income more equal - more difference (quadratic term) | 0.0011 | 0.0002 | 4.6654 | 0 |
| Highest Education 2 (ref. 1) | -0.0809 | 0.0062 | -13.1540 | 0 |
| Highest Education 3 (ref. 1) | -0.1574 | 0.0080 | -19.6238 | 0 |
| Highest Education 4 (ref. 1) | -0.2195 | 0.0069 | -31.9395 | 0 |
| Highest Education 5 (ref. 1) | -0.1845 | 0.0083 | -22.1876 | 0 |
| Highest Education 6 (ref. 1) | -0.2520 | 0.0073 | -34.5620 | 0 |
| Highest Education 7 (ref. 1) | -0.2920 | 0.0095 | -30.7410 | 0 |
| Highest Education 8 (ref. 1) | -0.3404 | 0.0075 | -45.5373 | 0 |
| Scales encoded in 10 steps | 0.0008 | 0.0009 | 0.9665 | 0.3338 |
| frequency of attendance of religious services | 0.025683 | 0.001087 | 23.63801 | 0 |


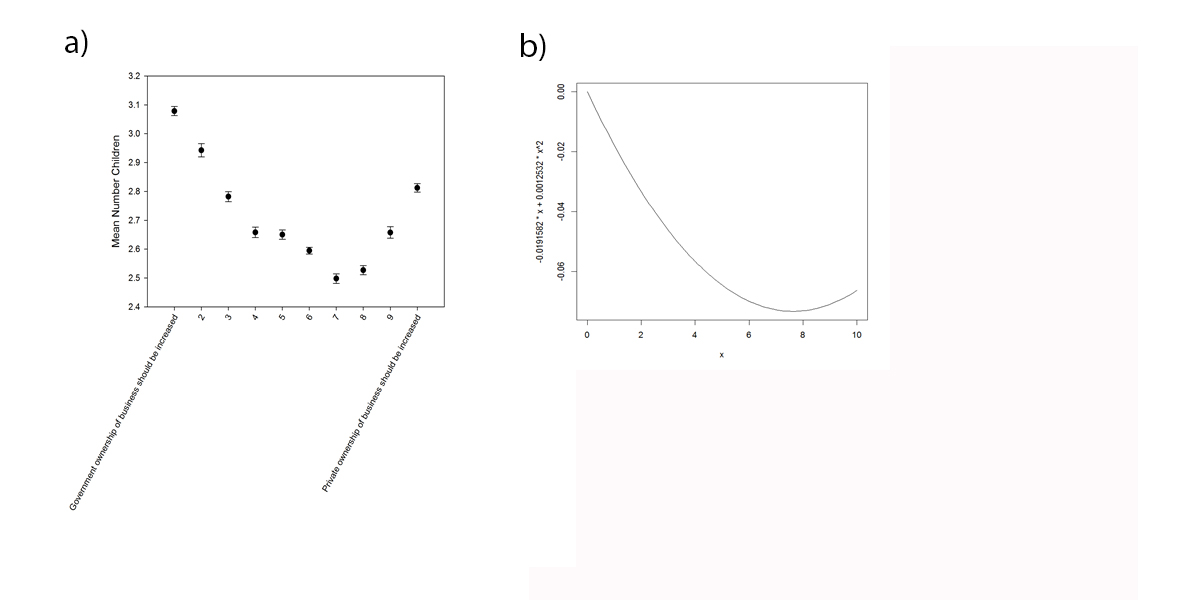


**Figure S4)** WVS data set: attitude towards “private- state - ownership” a) mean number of children, standard errors; b) quadratic model.

**Table S11)** WVS: Linear mixed model “private state ownership”

|  | **Value** | **Std.Error** | **t-value** | **p-value** |
| --- | --- | --- | --- | --- |
| (Intercept) | 0.7235 | 0.0334 | 21.6738 | 0 |
| Age | 0.0079 | 0.0002 | 44.2675 | 0 |
| Sex female (ref. male) | -0.0238 | 0.0036 | -6.5737 | 0 |
| gonverement influence increase - decrease (linear term) | -0.0192 | 0.0026 | -7.3571 | 0 |
| gonverement influence increase (quadratic term) | 0.0013 | 0.0002 | 5.4295 | 0 |
| Highest Education 2 (ref. 1) | -0.0821 | 0.0063 | -13.0106 | 0 |
| Highest Education 3 (ref. 1) | -0.1573 | 0.0082 | -19.1134 | 0 |
| Highest Education 4 (ref. 1) | -0.2189 | 0.0070 | -31.1759 | 0 |
| Highest Education 5 (ref. 1) | -0.1869 | 0.0085 | -22.0327 | 0 |
| Highest Education 6 (ref. 1) | -0.2506 | 0.0074 | -33.7321 | 0 |
| Highest Education 7 (ref. 1) | -0.2909 | 0.0097 | -30.1084 | 0 |
| Highest Education 8 (ref. 1) | -0.3387 | 0.0076 | -44.5355 | 0 |
| Scales encoded in 10 steps | 0.0013 | 0.0009 | 1.4841 | 0.1378 |
| frequency of attendance of religious services | 0.026318 | 0.001108 | 23.74806 | 0 |


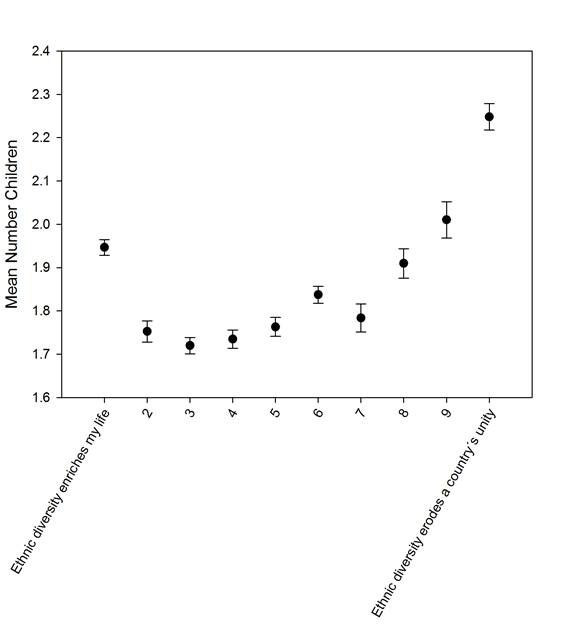


**Figure S5)** WVS data set: attitude towards “ethnic diversity” mean number of children, standard errors

**Table S12)** WVS: Linear mixed model “ethnic diversity”

|  | **Value** | **Std.Error** | **t-value** | **p-value** |
| --- | --- | --- | --- | --- |
| (Intercept) | 0.5583 | 0.0487 | 11.4568 | 0.0000 |
| Age | 0.0088 | 0.0004 | 22.9761 | 0.0000 |
| Sex female (ref. male) | -0.0310 | 0.0079 | -3.9229 | 0.0001 |
| ethnic diversity enriches life vs erodes life | -0.0008 | 0.0015 | -0.5503 | 0.5821 |
| Highest Education 2 (ref. 1) | -0.0855 | 0.0135 | -6.3121 | 0.0000 |
| Highest Education 3 (ref. 1) | -0.1525 | 0.0188 | -8.1024 | 0.0000 |
| Highest Education 4 (ref. 1) | -0.2339 | 0.0148 | -15.8361 | 0.0000 |
| Highest Education 5 (ref. 1) | -0.1985 | 0.0212 | -9.3864 | 0.0000 |
| Highest Education 6 (ref. 1) | -0.2682 | 0.0165 | -16.2581 | 0.0000 |
| Highest Education 7 (ref. 1) | -0.3140 | 0.0212 | -14.8324 | 0.0000 |
| Highest Education 8 (ref. 1) | -0.3416 | 0.0168 | -20.3745 | 0.0000 |
| Scales encoded in 10 steps | -0.0009 | 0.0020 | -0.4566 | 0.6480 |
| frequency of attendance of religious services | 0.0299 | 0.0025 | 11.8444 | 0.0000 |


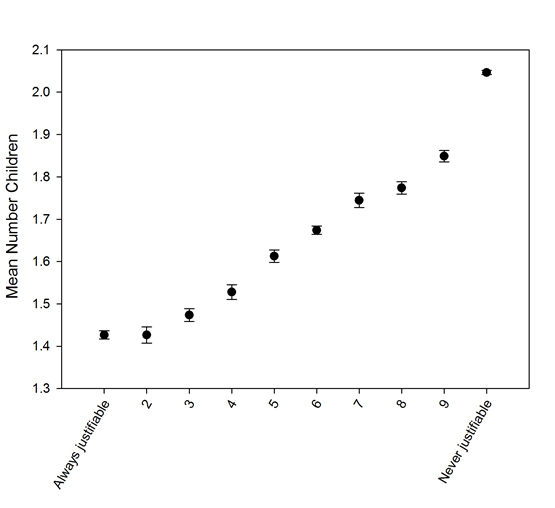


**Figure S6)** WVS data set: attitude towards “homosexuality” mean number of children, standard errors;

**Table S13)** WVS: Linear mixed model “homosexuality”

|  | **Value** | **Std.Error** | **t-value** | **p-value** |
| --- | --- | --- | --- | --- |
| (Intercept) | 0.6084 | 0.0330 | 18.4492 | 0.0000 |
| Age | 0.0077 | 0.0002 | 42.7309 | 0.0000 |
| Sex female (ref. male) | -0.0128 | 0.0037 | -3.4879 | 0.0005 |
| homo sexuality allways justifiable - never justifiable | 0.0082 | 0.0008 | 10.4093 | 0.0000 |
| Highest Education 2 (ref. 1) | -0.0857 | 0.0064 | -13.3107 | 0.0000 |
| Highest Education 3 (ref. 1) | -0.1643 | 0.0083 | -19.7327 | 0.0000 |
| Highest Education 4 (ref. 1) | -0.2198 | 0.0072 | -30.7073 | 0.0000 |
| Highest Education 5 (ref. 1) | -0.1864 | 0.0086 | -21.6838 | 0.0000 |
| Highest Education 6 (ref. 1) | -0.2492 | 0.0076 | -32.8880 | 0.0000 |
| Highest Education 7 (ref. 1) | -0.2834 | 0.0097 | -29.1103 | 0.0000 |
| Highest Education 8 (ref. 1) | -0.3341 | 0.0078 | -42.8037 | 0.0000 |
| Scales encoded in 10 steps | 0.0024 | 0.0009 | 2.7160 | 0.0066 |
| frequency of attendance of religious services | 0.0233 | 0.0011 | 20.3632 | 0.0000 |


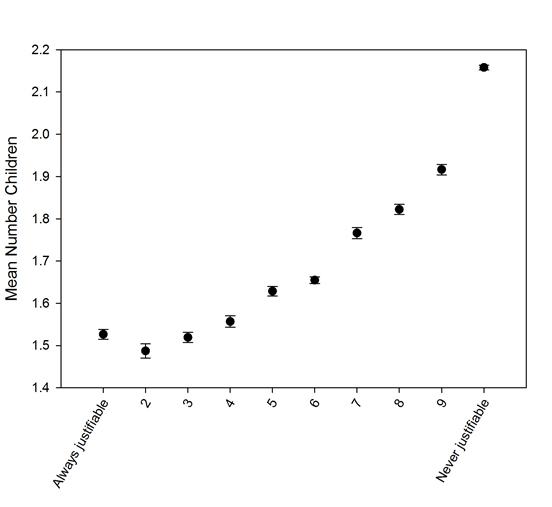


**Figure S7)** WVS data set: attitude towards “abortion” a) mean number of children, standard errors

**Table S14)** WVS: Linear mixed model “abortion”

|  | **Value** | **Std.Error** | **t-value** | **p-value** |
| --- | --- | --- | --- | --- |
| (Intercept) | 0.6072 | 0.0323 | 18.7747 | 0.0000 |
| Age | 0.0076 | 0.0002 | 43.2020 | 0.0000 |
| Sex female (ref. male) | -0.0165 | 0.0036 | -4.5972 | 0.0000 |
| abortion allways justifiable - never justifiable | 0.0104 | 0.0008 | 13.7928 | 0.0000 |
| Highest Education 2 (ref. 1) | -0.0823 | 0.0063 | -13.1606 | 0.0000 |
| Highest Education 3 (ref. 1) | -0.1600 | 0.0081 | -19.7105 | 0.0000 |
| Highest Education 4 (ref. 1) | -0.2162 | 0.0070 | -30.9013 | 0.0000 |
| Highest Education 5 (ref. 1) | -0.1789 | 0.0084 | -21.3783 | 0.0000 |
| Highest Education 6 (ref. 1) | -0.2450 | 0.0074 | -33.1700 | 0.0000 |
| Highest Education 7 (ref. 1) | -0.2798 | 0.0096 | -29.2890 | 0.0000 |
| Highest Education 8 (ref. 1) | -0.3294 | 0.0076 | -43.3066 | 0.0000 |
| Scales encoded in 10 steps | 0.0021 | 0.0009 | 2.4160 | 0.0157 |
| frequency of attendance of religious services | 0.0216 | 0.0011 | 19.2679 | 0.0000 |

**Table S15)** SHARE: Linear mixed model with number of children regressing on political orientation included as factor (most right as reference), age, sex, education (lowest education level 1 as reference), and scales of income on basis a Poisson error structure, with country as random factor.

|  | **Value** | **Std.Error** | **t-value** | **p-value** |
| --- | --- | --- | --- | --- |
| (Intercept) | 0.864 | 0.042 | 20.553 | p < 0.0001 |
| Age | 0.001 | 0.000 | 5.369 | p < 0.0001 |
| Sex female (ref. male) | -0.001 | 0.005 | -0.234 | 0.815 |
| Self Positioning left 0 (ref. right 10) | -0.049 | 0.016 | -3.000 | 0.003 |
| Self Positioning 1 (ref. right 10) | -0.080 | 0.023 | -3.489 | 0.001 |
| Self Positioning 2 (ref. right 10) | -0.040 | 0.017 | -2.307 | 0.021 |
| Self Positioning 3 (ref. right 10) | -0.037 | 0.015 | -2.472 | 0.013 |
| Self Positioning 4 (ref. right 10) | -0.045 | 0.015 | -3.025 | 0.003 |
| Self Positioning 5 (ref. right 10) | -0.039 | 0.013 | -3.067 | 0.002 |
| Self Positioning 6 (ref. right 10) | -0.034 | 0.015 | -2.268 | 0.023 |
| Self Positioning 7 (ref. right 10) | -0.013 | 0.015 | -0.859 | 0.390 |
| Self Positioning 8 (ref. right 10) | -0.009 | 0.015 | -0.59 | 0.555 |
| Self Positioning 9 (ref. right 10) | -0.024 | 0.022 | -1.072 | 0.284 |
| Highest Education 2 (ref. 1) | -0.090 | 0.014 | -6.283 | p < 0.0001 |
| Highest Education 3 (ref. 1) | -0.202 | 0.015 | -13.757 | p < 0.0001 |
| Highest Education 4 (ref. 1) | -0.255 | 0.015 | -17.537 | p < 0.0001 |
| Highest Education 5 (ref. 1) | -0.291 | 0.019 | -15.401 | p < 0.0001 |
| Highest Education 6 (ref. 1) | -0.272 | 0.015 | -18.194 | p < 0.0001 |
| Highest Education 7 (ref. 1) | -0.263 | 0.030 | -8.790 | p < 0.0001 |
| Household Income Percentiles | 0.011 | 0.001 | 11.656 | p < 0.0001 |
| DF | 55215 |  |  |  |
| Random factors | Intercept Country | Residuals |  |  |
| StdDev: | 0.1196945 | 0.8899632 |  |  |

**Tables S16-S20)** Linear mixed model separately for each ~ 10 year-period of the GSS, with number of children regressing on political orientation included as factor (most conservative as reference), age, sex, education (lowest education level “Lower than High School” as reference), and scales of income on basis a Poisson error structure, with year of survey and religious denomination as random factors.

| **Table S16) GSS: 1972-1979** | | | | | | | |  |  |  |  | |  | |  |
| --- | --- | --- | --- | --- | --- | --- | --- | --- | --- | --- | --- | --- | --- | --- | --- |
|  |  | | **Value** | |  | | **Std.Error** | | | | | **t-value** | | **p-value** | |
|  | (Intercept) | | 1.894 | |  | | 0.275 | | | | | 6.887 | | 0.000 | |
|  | Age | | -0.019 | |  | | 0.003 | | | | | -5.843 | | 0.000 | |
|  | Sex female (ref. male) | | -0.125 | |  | | 0.055 | | | | | -2.289 | | 0.022 | |
|  | extremly liberal ( ref.: extremly conservative) | | 0.497 | |  | | 0.233 | | | | | 2.134 | | 0.033 | |
|  | liberal ( ref.: extremly conservative) | | 0.388 | |  | | 0.186 | | | | | 2.092 | | 0.037 | |
|  | slightly liberal ( ref.: extremly conservative) | | 0.359 | |  | | 0.184 | | | | | 1.955 | | 0.051 | |
|  | moderate ( ref.: extremly conservative) | | 0.511 | |  | | 0.173 | | | | | 2.958 | | 0.003 | |
|  | slightly conservative ( ref.: extremly conservative) | | 0.474 | |  | | 0.176 | | | | | 2.687 | | 0.007 | |
|  | conservative( ref.: extremly conservative) | | 0.535 | |  | | 0.179 | | | | | 2.996 | | 0.003 | |
|  | High School (ref.: Lt High School) | | -0.207 | |  | | 0.051 | | | | | -4.096 | | 0.000 | |
|  | Junior College (ref.: Lt High School) | | -0.358 | |  | | 0.200 | | | | | -1.791 | | 0.074 | |
|  | Bachelor (ref.: Lt High School) | | -0.308 | |  | | 0.093 | | | | | -3.306 | | 0.001 | |
|  | Graduate (ref.: Lt High School) | | -0.188 | |  | | 0.106 | | | | | -1.780 | | 0.075 | |
|  | income | | -0.012 | |  | | 0.008 | | | | | -1.454 | | 0.146 | |
|  | DF | | 924 | |  | |  | | | | |  | |  | |
|  |  |  | |  | |  | | | | |  | |  | |  |
| **Table S17) GSS: 1980-1989** | |  | |  | |  | | | | |  | |  | |  |
|  |  | **Value** | |  | | **Std.Error** | | | | | **t-value** | | **p-value** | |  |
|  | (Intercept) | 1.635 | |  | | 0.166 | | | | | 9.831 | | 0.000 | |  |
|  | Age | -0.005 | |  | | 0.002 | | | | | -2.484 | | 0.013 | |  |
|  | Sex female (ref. male) | 0.030 | |  | | 0.035 | | | | | 0.834 | | 0.405 | |  |
|  | extremly liberal ( ref.: extremly conservative) | -0.073 | |  | | 0.144 | | | | | -0.511 | | 0.609 | |  |
|  | liberal ( ref.: extremly conservative) | -0.226 | |  | | 0.096 | | | | | -2.367 | | 0.018 | |  |
|  | slightly liberal ( ref.: extremly conservative) | -0.234 | |  | | 0.094 | | | | | -2.496 | | 0.013 | |  |
|  | moderate ( ref.: extremly conservative) | -0.152 | |  | | 0.080 | | | | | -1.889 | | 0.059 | |  |
|  | slightly conservative ( ref.: extremly conservative) | -0.212 | |  | | 0.086 | | | | | -2.472 | | 0.014 | |  |
|  | conservative( ref.: extremly conservative) | -0.168 | |  | | 0.086 | | | | | -1.954 | | 0.051 | |  |
|  | High School (ref.: Lt High School) | -0.131 | |  | | 0.038 | | | | | -3.460 | | 0.001 | |  |
|  | Junior College (ref.: Lt High School) | -0.192 | |  | | 0.106 | | | | | -1.807 | | 0.071 | |  |
|  | Bachelor (ref.: Lt High School) | -0.341 | |  | | 0.066 | | | | | -5.135 | | 0.000 | |  |
|  | Graduate (ref.: Lt High School) | -0.284 | |  | | 0.073 | | | | | -3.879 | | 0.000 | |  |
|  | income | -0.004 | |  | | 0.006 | | | | | -0.729 | | 0.466 | |  |
|  | DF | 1460 | |  | |  | | | | |  | |  | |  |
|  |  |  | |  | |  | | | | |  | |  | |  |
| **Table S18) GSS: 1990-1999** | |  | |  | |  | | | | |  | |  | |  |
|  |  | **Value** | |  | | **Std.Error** | | | | | **t-value** | | **p-value** | |  |
|  | (Intercept) | 0.705 | |  | | 0.159 | | | | | 4.429 | | P < 0.0001 | |  |
|  | Age | 0.009 | |  | | 0.002 | | | | | 4.703 | | P < 0.0001 | |  |
|  | Sex female (ref. male) | 0.002 | |  | | 0.032 | | | | | 0.052 | | 0.959 | |  |
|  | extremly liberal ( ref.: extremly conservative) | -0.034 | |  | | 0.137 | | | | | -0.246 | | 0.806 | |  |
|  | liberal ( ref.: extremly conservative) | -0.034 | |  | | 0.086 | | | | | -0.393 | | 0.694 | |  |
|  | slightly liberal ( ref.: extremly conservative) | -0.033 | |  | | 0.082 | | | | | -0.404 | | 0.686 | |  |
|  | moderate ( ref.: extremly conservative) | -0.074 | |  | | 0.072 | | | | | -1.015 | | 0.310 | |  |
|  | slightly conservative ( ref.: extremly conservative) | -0.049 | |  | | 0.076 | | | | | -0.643 | | 0.520 | |  |
|  | conservative( ref.: extremly conservative) | -0.022 | |  | | 0.076 | | | | | -0.289 | | 0.773 | |  |
|  | High School (ref.: Lt High School) | -0.184 | |  | | 0.043 | | | | | -4.294 | | 0.000 | |  |
|  | Junior College (ref.: Lt High School) | -0.255 | |  | | 0.075 | | | | | -3.423 | | 0.001 | |  |
|  | Bachelor (ref.: Lt High School) | -0.400 | |  | | 0.057 | | | | | -7.021 | | P < 0.0001 | |  |
|  | Graduate (ref.: Lt High School) | -0.406 | |  | | 0.067 | | | | | -6.051 | | P < 0.0001 | |  |
|  | income | -0.006 | |  | | 0.006 | | | | | -1.126 | | 0.260 | |  |
|  | DF | 1477 | |  | |  | | | | |  | |  | |  |
|  |  |  | |  | |  | | | | |  | |  | |  |
| **Table S19) GSS: 2000-2009** | |  | |  | |  | | | | |  | |  | |  |
|  |  | **Value** | |  | | **Std.Error** | | | | | **t-value** | | **p-value** | |  |
|  | (Intercept) | 0.563 | |  | | 0.182 | | | | | 3.100 | | 0.002 | |  |
|  | Age | 0.010 | |  | | 0.002 | | | | | 4.739 | | 0.000 | |  |
|  | Sex female (ref. male) | -0.065 | |  | | 0.035 | | | | | -1.872 | | 0.061 | |  |
|  | extremly liberal ( ref.: extremly conservative) | -0.323 | |  | | 0.147 | | | | | -2.192 | | 0.029 | |  |
|  | liberal ( ref.: extremly conservative) | -0.086 | |  | | 0.093 | | | | | -0.923 | | 0.356 | |  |
|  | slightly liberal ( ref.: extremly conservative) | -0.153 | |  | | 0.092 | | | | | -1.668 | | 0.096 | |  |
|  | moderate ( ref.: extremly conservative) | -0.115 | |  | | 0.079 | | | | | -1.459 | | 0.145 | |  |
|  | slightly conservative ( ref.: extremly conservative) | -0.123 | |  | | 0.085 | | | | | -1.443 | | 0.149 | |  |
|  | conservative( ref.: extremly conservative) | -0.111 | |  | | 0.082 | | | | | -1.346 | | 0.178 | |  |
|  | High School (ref.: Lt High School) | -0.120 | |  | | 0.056 | | | | | -2.143 | | 0.032 | |  |
|  | Junior College (ref.: Lt High School) | -0.147 | |  | | 0.078 | | | | | -1.876 | | 0.061 | |  |
|  | Bachelor (ref.: Lt High School) | -0.250 | |  | | 0.068 | | | | | -3.657 | | 0.000 | |  |
|  | Graduate (ref.: Lt High School) | -0.351 | |  | | 0.077 | | | | | -4.572 | | 0.000 | |  |
|  | income | -0.005 | |  | | 0.006 | | | | | -0.780 | | 0.435 | |  |
|  | DF | 1492 | |  | |  | | | | |  | |  | |  |
|  |  |  | |  | |  | | | | |  | |  | |  |
| **Table S20) GSS: 2010-2014** | |  | |  | |  | | | | |  | |  | |  |
|  |  | **Value** | |  | | **Std.Error** | | | | | **t-value** | | **p-value** | |  |
|  | (Intercept) | 0.874 | |  | | 0.226 | | | | | 3.861 | | 0.000 | |  |
|  | Age | 0.009 | |  | | 0.003 | | | | | 3.062 | | 0.002 | |  |
|  | Sex female (ref. male) | 0.009 | |  | | 0.048 | | | | | 0.178 | | 0.858 | |  |
|  | extremly liberal ( ref.: extremly conservative) | -0.257 | |  | | 0.174 | | | | | -1.476 | | 0.140 | |  |
|  | liberal ( ref.: extremly conservative) | -0.266 | |  | | 0.118 | | | | | -2.244 | | 0.025 | |  |
|  | slightly liberal ( ref.: extremly conservative) | -0.068 | |  | | 0.114 | | | | | -0.593 | | 0.553 | |  |
|  | moderate ( ref.: extremly conservative) | -0.145 | |  | | 0.094 | | | | | -1.535 | | 0.125 | |  |
|  | slightly conservative ( ref.: extremly conservative) | -0.097 | |  | | 0.104 | | | | | -0.934 | | 0.350 | |  |
|  | conservative( ref.: extremly conservative) | -0.064 | |  | | 0.098 | | | | | -0.655 | | 0.513 | |  |
|  | High School (ref.: Lt High School) | -0.272 | |  | | 0.076 | | | | | -3.574 | | 0.000 | |  |
|  | Junior College (ref.: Lt High School) | -0.323 | |  | | 0.122 | | | | | -2.657 | | 0.008 | |  |
|  | Bachelor (ref.: Lt High School) | -0.341 | |  | | 0.091 | | | | | -3.759 | | 0.000 | |  |
|  | Graduate (ref.: Lt High School) | -0.439 | |  | | 0.098 | | | | | -4.490 | | 0.000 | |  |
|  | income | -0.019 | |  | | 0.008 | | | | | -2.264 | | 0.024 | |  |
|  | DF | 803 | |  | |  | | | | |  | |  | |  |

**Table S21)** GSS: General models of number of children regressing on political orientation included as factor (most conservative as reference), age, sex, education (lowest education level “Lower than High School” as reference), income on basis a Poisson error structure, for each survey separately: only estimates for political orientation are shown (significant estimates in bold).

|  | **extremely liberal ( ref.: extremely conservative)** | **liberal ( ref.: extremely conservative)** | **slightly liberal ( ref.: extremely conservative)** | **moderate ( ref.: extremely conservative)** | **slightly conservative ( ref.: extremely conservative)** | **conservative( ref.: extremley conservative)** |
| --- | --- | --- | --- | --- | --- | --- |
| #1974 | -0.293605 | -0.068126 | 0.069365 | 0.277691 | 0.162146 | 0.136495 |
| #1975 | 0.27753 | **0.59058*** | **0.53951.** | **0.68846*** | **0.66589*** | **0.66851*** |
| #1976 | -0.203771 | 0.046088 | -0.066117 | 0.042283 | 0.184159 | 0.034498 |
| #1977 | 0.20396 | 0.382827 | 0.115053 | 0.327281 | 0.445436 | **0.505662*** |
| #1978 | 1.155718 | 0.412984 | 0.705701 | 0.747202 | 0.703183 | 0.830104 |
| #1979 | **1.15571.** | 0.412984 | 0.705701 | 0.747202 | 0.703183 | 0.830104 |
| #1980 | -0.204455 | **-0.507127.** | -0.150791 | 0.011676 | 0.079424 | 0.049688 |
| #1982 | 0.083393 | -0.040854 | -0.08952 | -0.112903 | 0.010678 | -0.157589 |
| #1983 |  | -0.267332 | 0.122042 | -0.004331 | -0.207987 | -0.16028 |
| #1984 | 0.117802 | -0.337987 | -0.354931 | -0.217892 | -0.247685 | -0.047376 |
| #1985 | 0.549095 | 0.323659 | **0.461792.** | 0.384264 | 0.277916 | 0.297139 |
| #1986 | -0.0160358 | **-0.4047227.** | -0.2387892 | -0.1247713 | -0.290804 | -0.1838176 |
| #1987 | -0.189813 | -0.134466 | -0.250969 | -0.147125 | -0.306791 | -0.139138 |
| #1988 | -0.226178 | -0.059171 | -0.337749 | **-0.331119.** | -0.299091 | -0.292599 |
| #1989 | 0.123391 | -0.19547 | -0.126842 | -0.054699 | -0.082631 | -0.225162 |
| #1990 | -0.014641 | -0.1455 | -0.185973 | 0.01042 | -0.080218 | -0.012674 |
| #1991 | 0.361116 | -0.390863 | -0.032051 | 0.010034 | 0.069534 | 0.014144 |
| #1992 | 0.361116 | -0.390863 | -0.032051 | 0.010034 | 0.069534 | 0.014144 |
| #1993 | -0.059351 | 0.275203 | 0.266917 | 0.313645 | 0.400375 | 0.27928 |
| #1994 | 0.075336 | -0.090138 | -0.043189 | -0.081037 | -0.048708 | 0.033623 |
| #1995 | 0.075336 | -0.090138 | -0.043189 | -0.081037 | -0.048708 | 0.033623 |
| #1996 | -0.14867 | 0.031271 | 0.100944 | -0.034667 | 0.005506 | -0.03907 |
| #1997 | -0.14867 | 0.031271 | 0.100944 | -0.034667 | 0.005506 | -0.03907 |
| #1999 | -0.14867 | 0.031271 | 0.100944 | -0.034667 | 0.005506 | -0.03907 |
| #2000 | -0.319661 | -0.241004 | -0.234962 | -0.168145 | -0.150151 | -0.278349 |
| #2002 | -0.499554. | -0.247267 | -0.264895 | -0.203246 | -0.232982 | -0.231641 |
| #2003 | -0.499554. | -0.247267 | -0.264895 | -0.203246 | -0.232982 | -0.231641 |
| #2004 | **-1.492248*** | 0.266579 | 0.111443 | 0.177038 | 0.21448 | 0.39768 |
| #2006 | **-0.797222***** | -0.187963 | -0.197999. | -0.092452 | -0.074716 | -0.082231 |
| #2008 | -0.0404256 | 0.0144136 | 0.0160861 | -0.0558103 | -0.0720023 | -0.0575171 |
| #2010 | -0.19872 | **-0.41319**** | -0.21213 | -0.21757 | **-0.25622.** | **-0.2413.** |
| #2012 | -0.2919 | -0.2459 | -0.1241 | -0.1264 | -0.1345 | -0.1473 |
| #2014 | **-0.4192091*** | -0.0603571 | 0.0441961 | 0.0007482 | 0.0857561 | -0.0313393 |


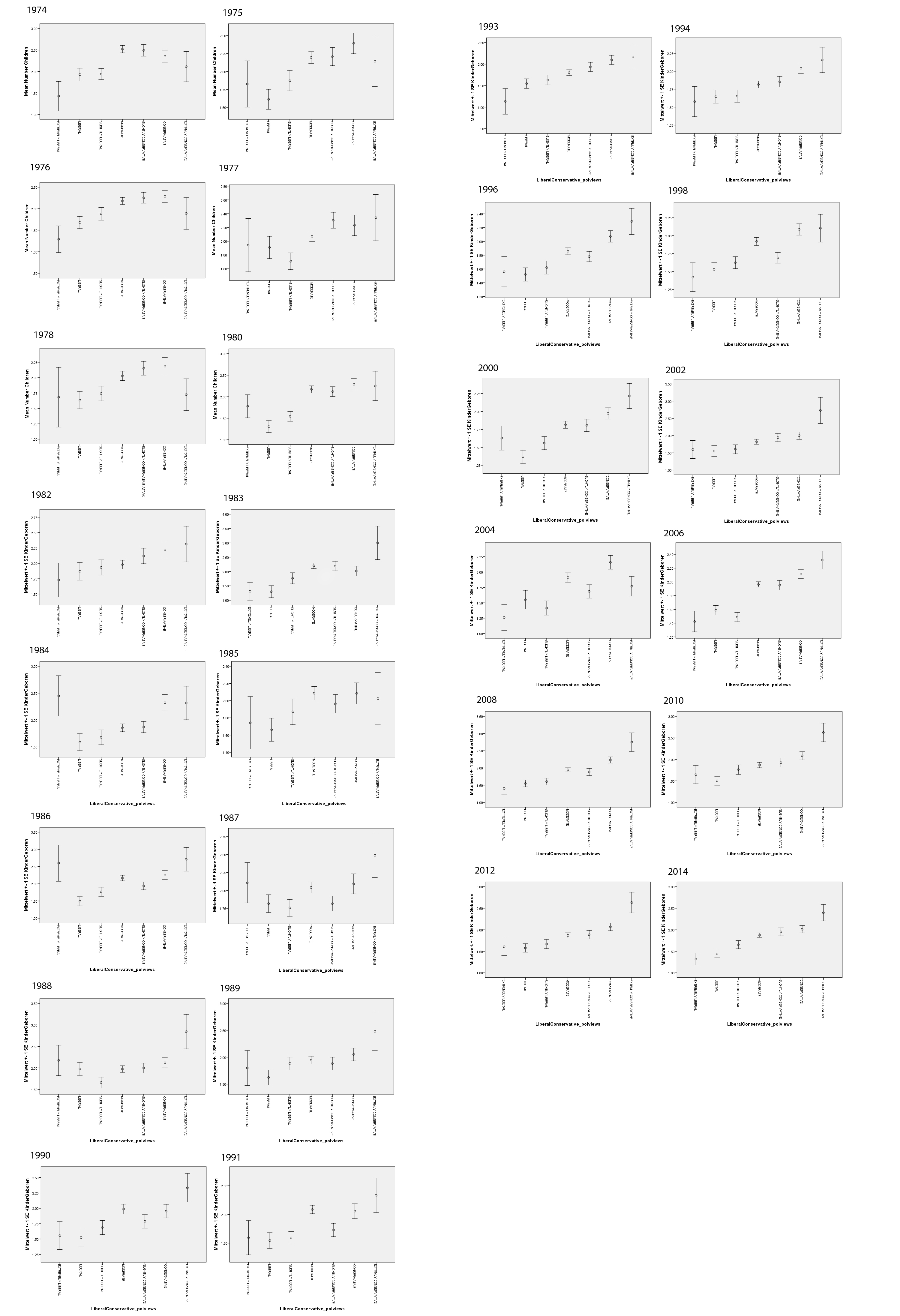


**Figure S8)** GSS: Political orientation and mean (±SE) number of children separately for each year.

**Table S22)** GSS: Overall model including interactions between education and religious attendance.

|  | **Value** | **Std.Error** | **t-value** | **p-value** |
| --- | --- | --- | --- | --- |
| (Intercept) | 0.96570 | 0.07338 | 13.15947 | 0.00000 |
| Age | 0.00315 | 0.00089 | 3.54879 | 0.00040 |
| Sex female (ref. male) | -0.03279 | 0.01432 | -2.29038 | 0.02200 |
| extremely liberal ( ref.: extremely conservative) | -0.16954 | 0.05643 | -3.00452 | 0.00270 |
| liberal ( ref.: extremely conservative) | -0.07718 | 0.03976 | -1.94133 | 0.05220 |
| slightly liberal ( ref.: extremely conservative) | -0.06435 | 0.03919 | -1.64180 | 0.10070 |
| moderate ( ref.: extremely conservative) | -0.02618 | 0.03500 | -0.74789 | 0.45450 |
| slightly conservative ( ref.: extremely conservative) | -0.02339 | 0.03696 | -0.63282 | 0.52690 |
| conservative( ref.: extremley conservative) | -0.02146 | 0.03697 | -0.58058 | 0.56150 |
| High School (ref.: Lt High School) | -0.22462 | 0.03084 | -7.28395 | 0.00000 |
| Junior College (ref.: Lt High School) | -0.40812 | 0.06392 | -6.38530 | 0.00000 |
| Bachelor (ref.: Lt High School) | -0.56613 | 0.04465 | -12.68008 | 0.00000 |
| Graduate (ref.: Lt High School) | -0.50176 | 0.04987 | -10.06222 | 0.00000 |
| Income | 0.01139 | 0.00514 | 2.21454 | 0.02680 |
| Number Religous Services | -0.00407 | 0.00241 | -1.68621 | 0.09180 |
| High School (ref.: Lt High School) : Number Religous Services | 0.01147 | 0.00617 | 1.85817 | 0.06320 |
| Junior College (ref.: Lt High School): Number Religous Services | 0.03578 | 0.01232 | 2.90398 | 0.00370 |
| Bachelor (ref.: Lt High School): Number Religous Services | 0.04456 | 0.00863 | 5.16483 | 0.00000 |
| Graduate (ref.: Lt High School): Number Religous Services | 0.02572 | 0.00961 | 2.67682 | 0.00740 |
| N | 10764 |  |  |  |
| Survey Year | (Intercept) | Residual |  |  |
| StdDev: | 0.07759 | 1.07806 |  |  |
